# How much improvement can satisfy patients? Exploring patients’ satisfaction 3 years after total knee arthroplasty

**DOI:** 10.1186/s13018-021-02514-2

**Published:** 2021-06-17

**Authors:** Xiao Yu Fan, Jin Hui Ma, Xinjie Wu, Xin Xu, Lijun Shi, Tengqi Li, Peixu Wang, Chengxin Li, Zhizhuo Li, Qing Yu Zhang, Wei Sun

**Affiliations:** 1 Peking University Health Science Center, China-Japan Friendship School of Clinical Medicine, Beijing, 100029 China; 2 Orthopedics Department, China-Japan Friendship Hospital, Beijing, 100029 China; 3 Peking Union Medical College, China-Japan Friendship School of Clinical Medicine, Beijing, 100029 China; 4 Department of Orthopedics, Shandong Provincial Hospital Affiliated to Shandong First Medical University, No. 324, Road Jing Wu Wei Qi, Jinan, 250021 Shandong China

**Keywords:** Satisfaction, Total knee arthroplasty, Minimal clinically important difference, Minimum important change, Radiological parameter

## Abstract

**Background:**

Despite the innovations in total knee arthroplasty (TKA), there is still a subset of patients who do not acquire significant relief or expected satisfaction after primary TKA. However, this subgroup of patients still gains improvements more or less in terms of objective or quantified assessments after the procedure. The purpose of our study is to explore the factors that correlate with patients’ satisfaction and identify minimal clinically important difference (MCID) and minimum important change (MIC) in clinical parameters.

**Methods:**

We conducted a retrospective study of 161 patients diagnosed with osteoarthritis who underwent unilateral total knee arthroplasty from January 2017 to December 2017. We collected the following parameters: body mass index (BMI), duration of disease, education level, depression state, preoperative flexion contracture angle of knee, HSS scores, 11-point NRS scores, and radiological parameters (preoperative minimal joint space width and varus angle of knee). The satisfaction was graded by self-reported scores in percentage (0–100).

**Results:**

We revealed that 80.8% of patients were satisfied 3 years overall after primary TKA. HSS score change, NRS-Walking score change, age, and pre-mJSW showed significant difference between satisfied and dissatisfied group. The varus angle change revealed statistical significance according to the levels of satisfaction. Simple linear regression identified the MCID for HSS score to be 5.41 and for the NRS-Walking to be 1.24. The receiver operating characteristics (ROC) curve identified the MIC for HSS score to be 25.5 and for the NRS-Walking score to be 6.5.

**Conclusions:**

In summary, we identified several factors that correlated with patients’ satisfaction independently after TKA in a long term. In addition, we revealed the minimal clinically important difference (MCID) and minimum important change (MIC) for HSS and NRS score in these patients.

**Supplementary Information:**

The online version contains supplementary material available at 10.1186/s13018-021-02514-2.

## Background

Knee osteoarthritis has become an extraordinarily common disease in modern societies worldwide with a nearly standardized annual incidence of 24/10,000 population [1]. The degenerative diseases of the knee had significantly reduced the life quality of the aged and brought about a series of complications due to the long-term condition of immobilization, such as venous thrombus embolism and hypostatic pneumonia. Total knee arthroplasty (TKA), as one of the greatest inventions in twentieth century, has become a radical treatment for late-staged knee osteoarthritis. This revolutionary procedure is capable of ameliorating the pain and improving the function in the majority of patients [2, 3].

Despite the innovations of TKA, some patients still report unsatisfactory experience or inconsistently self-reported outcomes after the operation. Furthermore, if we compare the numerical values in quantified parameters before and after the surgery, patients with different outcomes will often be associated with different alterations. In order to explore the relation of patients’ outcomes to the changes in perioperative quantified indexes, we tend to find out the minimal clinically important difference (MCID) according to the level of patients’ satisfaction after primary TKA. Defined by the previous studies, MCID revealed the difference in the mean change in the score between patients with “no” improvement compared to those with “a little” improvement according to the anchor question [4]. The point of defining this concept is to reveal the smallest improvement that a patient would describe as clinically important. And then it should be an important metric that readers or clinician-scientists consider when they evaluate therapeutic claims in clinical researches.

According to the previous studies, up to 10–20% of patients would express overall dissatisfaction with their primary total knee arthroplasty [5–7]. Furthermore, previous researchers found out that incomplete pain relief and limited functional recovery were the two leading causes of discontented self-reported outcomes [7]. On the other hand, besides self-reported outcomes or satisfaction rate, objective measurements before and after the procedure can be viewed as a reflection as well, such as perioperative HSS score, NRS or VAS, WOMAC, and even radiological parameters. The significance of objective assessments lies on eliminating some of the subjective factors since former studies had pointed out that patients’ satisfaction could be a mixed parameter that might be largely influenced by either the evaluation of the hospitalized care or the process by which the “service” was delivered [8, 9]. For patients with dissatisfaction or below expectation, the feedback of the treatments also varied in terms of physiological parameters or improvements in physical findings (e.g., HSS score, radiological changes).

Stratford et al. defined the MCID as ‘the smallest change that is meaningful and important to patients’ [10]. As Ostelo R. once indicated in his study, estimating the MCID of relevant outcomes enables a comparison between interventions on patient level and can contribute to the relevance and interpretability of change scores [11]. The appropriate clinical interpretation of changes on a numerical scale must consider not only statistical significance, but also whether the observed changes are clinically meaningful to patients.

The purpose of our study is to explore the factors that correlate with patients’ satisfaction and identify minimal clinically important difference (MCID) and minimum important change (MIC) in clinical parameters.

## Materials and method

### Population selection and characteristic

Patients for this study were identified retrospectively from a compiled arthroplasty cohort held at our clinical centre who underwent unilateral total knee arthroplasty(TKA) for knee osteoarthritis (OA) from January 2017 to December 2017. To exclude the bias caused by different surgeons’ technical factors, patients’ procedures were performed by the same attending group. Inclusion criteria were as follows: (1) diagnosed as osteoarthritis of the knee; (2) primary TKA was scheduled without any surgical operations before; (3) the availability of peri-operative X-rays of a standard anterior and lateral position of the knees and full-length radiographs of the lower limbs as well. Exclusion criteria were as follows: (1) inflammatory joint diseases, septic arthritis or tuberculous arthritis; (2) osteonecrosis, fractures, or bone tumor of the target knee(s) which requires TKA; (3) unrelated death or unwillingness to answer questions during follow-up.

### Date collection

Patients completed a questionnaire without any implication from the medical unit at baseline and 3-year follow-up section. The following preoperative information were collected: age, side, sex, disease duration, body mass index (BMI), education level, and radiographic findings including the minimal joint space width (pre-mJSW), preoperative flexion contracture of knee (FCK), and varus/valgus angle of knee (VAK). Postoperative information were collected as follows: satisfaction rate (percentage) and complications (deep vein thrombosis, pulmonary embolism, incision infection or disunion, or any readmission related to the procedure). The following parameters were collected both preoperatively and postoperatively: mental health, HSS score, 11-point NRS (rest and walking), and VAK in X-rays.

The patients’ education level was recorded according to the following pattern: “well-educated” (those with the degree of university or higher); “basically educated” (those with the degree of middle or high school); “poorly educated” (those with the degree of primary school or no experience of education).

The evaluation of patients’ mental health was assessed at baseline and 3 years after operation. Two conditions would be regarded as “depression state” in our study: (1) patients diagnosed with MDD (major depressive disorder) at baseline; (2) during the follow-up section, we applied the Hamilton Depression Rating Scale (HAM-D, 24-item) for the patients without the history of depression, and patients with total score greater than 8 were defined as “depression state” [12].

Digital photographs were taken of the target knee in the standard anterior and lateral position, and lower-limb full-length radiographs were applied for all patients pre- and postoperatively. All preoperative minimal joint space width (pre-mJSW) and radiological alignment readings were completed by three independent experienced observers who were blinded to patient demographics and outcomes. The radiological parameters were all measured by the PACS system (see Supplementary Material).

Postoperative function and pain improvements were measured by Hospital Special Surgery (HSS) score and numerical analog score (11-point NRS) in rest and walking. In addition, we absorbed the changes in the alignments after TKA as another aspect of objective parameters. The follow-up questionnaires must be answered by the patients themselves.

### Grade of satisfaction

Satisfaction was measured by asking the patients to rate their feedbacks towards the operation and postoperative rehabilitation after 3 years. The responses would be recorded in numerical form initially, but converted to 4-point scale eventually.

Patients’ satisfaction towards the overall procedures and rehabilitation was considered as the primary parameter for subjective outcomes. In our study, we divided the satisfaction rate into four grades: “very satisfied,” “satisfied,” “acceptable,” “disappointed.”

### Surgery procedures and implant materials

All TKA procedures were performed in the same operation room (OR 501) by the same attending group. In order to control the covariates in surgery, posterior-stabilized (PS) prosthesis was implanted in all patients. All the implants are made of Co-Cr-Mo alloy produced by the same corporation.

### Defining the MCID and MIC

We applied the anchor-based approach to establish the MCID in our study [13]. Anchor-based methods determine the MCID by associating the changes in the numerical scale of independent assessment of improvement with patient-reported subjective outcome(s) [14]. At the end of our follow-up questionnaire, one question would be asked: “Given the overall rehabilitation 3 years after TKA, how much will you rate your satisfaction if 100% means perfect or extremely satisfied”? The MCID was calculated as the difference in the mean change between patients with just “satisfied” group (81–90%) compared with those with just “acceptable” (61–80%) group.

In addition, we define the MIC (minimum important change) as the change in the score relative to the baseline for patients who reported “the minimal improvement” (“satisfied” group) based on a clinically meaningful reference measure according to the anchor question. Receiver operating characteristic (ROC) curve was used to identify threshold which is equivalent to the point achieving the maximal sensitivity and specificity in predicting the minimal improvement.

### Data analysis

Descriptive statistics were performed on all study data. Besides the 4-point scale satisfaction, patients were also divided into two groups based on whether they were satisfied (“very satisfied and “satisfied” group) or dissatisfied (“acceptable” and “disappointed” group). Unpaired Student’s *t* test and non-parametric Wilcoxon rank-sum (Mann–Whitney) test were used for continuous variables, and the chi-square test for categorical variables. Paired Student’s *t* test was applied for preoperative and postoperative assessments. One-way analysis of variance (ANOVA) with correction for multiple testing (Bonferroni) was used to compare means between groups. Variables were entered into a multiple ordinal logistic regression predicting the independent associations of them with patient's satisfaction. Variables tested included age, sex, BMI, HSS score change, NRS-walking change, pre-mJSW, and depression state.

Spearman’s rank correlation and Kendall’s rank correlation were used to identify the relevance between continuous and/or categorical variables. Simple linear regression analysis was used to identify the MCID, using the slope of the line for the change according to different level of satisfaction in the HSS and NRS score. The receiver operating characteristics (ROC) curve was used to define the MIC (threshold) that best discriminated (maximum sum of specificity and sensitivity of the model) between “satisfied” and “acceptable” group.

Statistical significance was set at p < 0.05 and all tests were 2-tailed. All statistical analyses and illustrations were done using Stata (version 15.1) software (StataCorp) and GraphPad Prism (version 8.0).

## Results

We retrospectively collected the baseline data of 180 patients who underwent TKA for osteoarthritis from January 2017 to December 2017 in total. After the completion of follow-up enquiry, 161 patients were enrolled in the final cohort (4 patients died of unrelated diseases, 6 patients were unable to answer questions themselves due to cognitive disability, 7 patients were lost to follow-up, and 2 patients refused to answer the questionnaire). Table 1 reports the baseline information of the final cohort.

**Table 1:** Preoperative baseline characteristics of the patients

	Total patients (n = 161)
Age (mean ± SD) (years)	49-89 (67.90 ± 7.26)
Sex (female/male)	124(77%)/37(23%)
BMI (mean ± SD) (kg/m^2^)	17.08–35.57 (26.23 ± 3.57)
Disease duration (mean ± SD) (years)	0.2–60 (10.75 ± 9.12)
NRS-Rest (Med; IQR)	0–7 (0; 2)
NRS-Walking (mean ± SD)	3–10 (7.88 ± 1.21)
Preoperative HSS score	33–71 (56.16 ± 7.77)
Preoperative minimal joint space width (Med; IQR) (mm)	0–10.46 (1.14; 3.81)

Continuous variables data: range (mean ± SD)

Non-normal distribution data: range (Med; IQR)

As the primary outcome after 3 years, the patients’ satisfaction ratings were recorded as follows: “very satisfied” group, n = 88 (54.7%); “satisfied” group, n = 42 (26. 1%); “acceptable” group, n = 25(15.5%); “disappointed” group, n = 6 (3.7%). In addition, we summarized the overall subjective outcomes as “satisfied group (very satisfied plus satisfied)” and “dissatisfied group (acceptable plus disappointed),” 80.8% (n = 130) of patients were satisfied 3 years after TKA on the whole. There were significant differences in HSS change, NRS-Walking change, age, and pre-mJSW between “satisfied” and “dissatisfied” groups (p < 0.05). BMI, sex(female proportion), NRS-Rest change, depression state, and preoperative flexion contracture of knee were not statistically significant between the two groups (Table 2). Besides numerical scale assessments, radiological parameter changes were found to be associated with different levels of satisfaction as well (Table 3). Significant improvements or corrections were observed in HSS score, NRS-walking score, and radiological parameter between baseline and 3-year postoperative evaluation (Table 4). After controlling for confounding variables, such as sex, BMI, depression, and disease duration, we found that age < 65 at baseline (odds ratio [OR], 1. 22; 95% confidence interval [CI] = 1.15–1.31; p < 0.05), HSS score change (OR, 1.06; 95% CI = 1.00–1.12; p < 0.05), NRS-walking change (OR, 2. 80; 95% CI = 2.04–3.85; p < 0.05), and pre-mJSW < 2 mm (OR, 4.61; 95% CI = 2.08–10.22; p < 0.05) were independently associated with higher patient’s satisfaction (Table 5).

**Table 2:** Features of the study population 3 years after total knee arthroplasty according to overall satisfaction

	Satisfied (N = 130) Mean (95% CI)	Dissatisfied (N = 31) Mean (95% CI)	P value
BMI (kg/m^2^)	26.34 (25.69–26.99)	25.79 (24.85–26.73)	N.S.S
HSS change	36.71(35.37–38.05)	27.92(24.80–31.04)	< 0.05
NRS-Walking change	7.75(7.55–7.94)	3.91(3.27–4.53)	< 0.05
NRS-Rest change	0 (1)^**#**^	1(2)^**#**^	N.S.S
	N(%)	N(%)	
Female	103(79.2%)	21(67.7%)	N.S.S
Age > 65 at baseline	75(57.7%)	26(83.9%)	< 0.05
BMI ≥ 25 kg/m^2^	86(66.2%)	23(76.7%)	N.S.S
Pre-mJSW < 2 mm	84(64.6%)	13(43.3%)	< 0.05
Depression state*	6(4.6%)	4(12.9%)	N.S.S
Preoperative FCK	92(70.8%)	24(77.4%)	N.S.S

*N.S.S* not statistical significance, *BMI* body mass index, *pre-mJSW* preoperative minimal joint space width, *FCK* flexion contracture of knee

*Fisher’s exact test

^**#**^Non-normal distribution data (Med, IQR)

**Table 3:** The changes in objective outcomes according to the level of satisfaction 3 years following TKA

Outcomes	All patients (mean, 95% CI)	Satisfaction(mean±SD)				p value
		Very satisfied	Satisfied	Acceptable	Disappointed	
HSS score change (N = 161)	34.94 (33.58–36.30)	39.15 ± 7.39	31.61 ± 5.76	29.02 ± 8.38	23.33 ± 8.12	< 0.05*
NRS-Walking change (N = 161)	7.38 (7.12–7.64)	7.94 ± 1.12	7.43 ± 0.91	6.36 ± 1.52	2.17 ± 0.79	< 0.05*
Varus angle change (N = 145)	6.22(5.40–7.03)	6.70 ± 4.08	5.76 ± 4.46	5.54 ± 4.70	3.41 ± 0.49	< 0.05*

*ANOVA

**Table 4:** Preoperative and 3-year postoperative HSS score, NRS-Walking score, and radiological findings for the study cohort

Outcomes	Preoperative		3 years after TKA		P value
	Mean (range)	SD	Mean (range)	SD	
HSS (n = 161)	56.16(33–71)	7.77	91.10(67–100)	5.07	< 0.05
NRS-Walking (n = 161)	7.88(3–10)	1.21	0*(0–6)	1*	< 0.05
Varus angle of the knee (n = 145)(°)	10.12(0–31.15)	6.01	3.56*(0–13.75)	3.45*	< 0.05

*Non-normal distribution data: median (range), IQR

**Table 5:** Multivariable logistic regression model results for patients’ satisfaction

Satisfaction	Odds ratio	Std. err.	z	P	95% conf. interval
Duration	0.993774	0.0201667	− 0.31	0.758	0.9550237–1.034097
Sex	1.299435	0.602238	0.57	0.572	0.5239084–3.22295
Age^a^	1.223833	0.0410019	6.03	0.000	1.146052–1.306892
BMI^b^	0.9811767	0.0556674	− 0.33	0.738	0.8779181–1.09658
Depression	1.531385	1.434111	0.46	0.649	0.2443158–9.598804
HSS change	1.059726	0.031152	1.97	0.047	1.000395–1.122576
NRS-Walking change	2.802223	0 .454463	6.35	0.000	2.039178–3.850792
Pre-mJSW^c^	4.609576	1.873051	3.76	0.000	2.078665–10.22204

^a^Age was calculated as binary variable according to age > 65 or ≤ 65 at baseline

^b^BMI (body mass index) was calculated as binary variable according to BMI ≥ 25 kg/m^2^ or < 25 kg/m^2^ at baseline

^c^Pre-mJSW (minimum joint space width) was calculated as binary variable according to pre-mJSW< 2 mm or ≥ 2 mm at baseline

Using Spearman’s rank correlation, referring to the magnitude of correlation efficacy by the study of Patrick Schober [15], we found significant correlation between satisfaction and change in HSS score (rho = 0.55, p < 0.05, n = 161), change in NRS-Walking score (rho = 0.51, p < 0.05, n = 161). On the other hand, the correlation between varus angle change and patients’ satisfaction was relatively weak (rho = 0.14, p > 0.05, n = 145). By Kendall’s rank correlation, we found an inverse correlation between satisfaction and education level (tau-b = − 0.21, p < 0. 05, n = 161) although the correlation was weak.

**Fig. 1:**
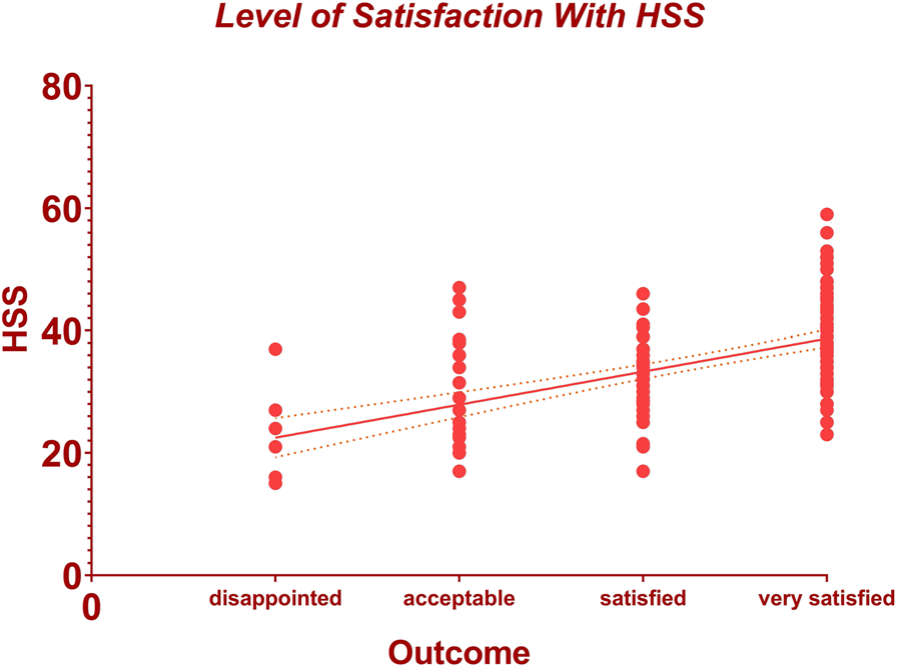
The correlation between the changes in HSS score and different levels of patient satisfaction

**Fig. 2:**
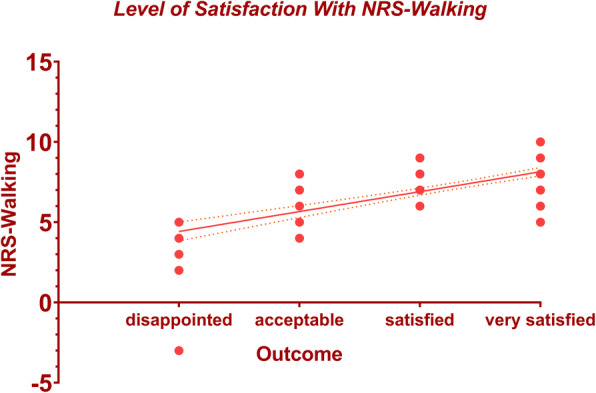
The correlation between the changes in NRS-Walking score and different levels of patient satisfaction

**Fig. 3:**
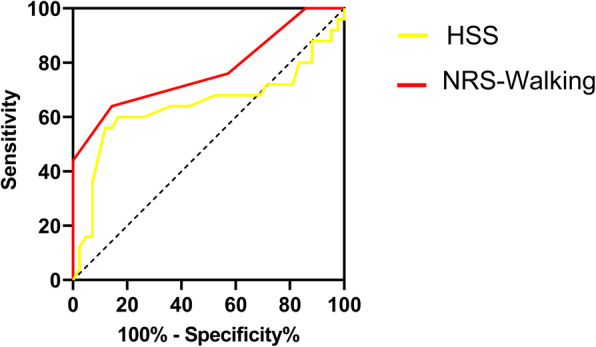
ROC curves revealing the MIC (minimum important change) from patients who just benefited minimal satisfaction for HSS and NRS-Walking scores

After adjusting for confounding variables (age, gender, BMI, depression state, and disease duration) between the groups (acceptable and satisfied), simple linear regression identified the MCID for HSS score to be 5.41 (95% CI 4.11–6.71, Adj. R^2^ = 0.30) and MCID for the NRS-Walking score to be 1.24 (95% CI 1.00–1.48, Adj. R^2^ = 0.41). Changes in HSS score with 95% CI according to the different levels of patient satisfaction were depicted in Fig. 1. Likewise, the correlation between the changes in NRS-Walking score and different levels of satisfaction was illustrated in Fig. 2. Using the receiver operating characteristics (ROC) curve, the MIC (threshold with maximal specificity and sensitivity)for the HSS score was 25.5 with an AUC of 0.65 (95% CI 0.49–0.80), and MIC for the NRS-Walking score was 6.5 with an AUC of 0.77 (95% CI 0.64–0.90) (Fig. 3).

## Discussion

The purpose of our study is to identify the relative factors to patients’ satisfaction 3 years after primary TKA and MIC, MCID for HSS and NRS score. The innovative values in this study are the revelation of association between patient’s satisfaction and their preoperative radiological parameter (varus angle of knee) and limb deformity. In our study, the overall satisfaction rate 3 years after the procedure was 80.8%, which was consistent with previous studies [3, 5, 16–18]. We demonstrated several factors independently associated with satisfaction: age, HSS score change, NRS-Walking change, and preoperative minimum joint space width. However, unlike several previous studies [7, 19], we found no significant correlation between depression and satisfaction. One possible explanation for this disaccordance is the difficulty in interpreting whether the psychological symptoms are the cause or effect of preoperative pain in the knee. In other words, preoperative knee pain and discomforts are likely to contribute significantly to preoperative depression or anxiety. Besides, there was no statistical difference between satisfied and dissatisfied group in terms of preoperative flexion contracture of knee although preoperative minimal joint space width showed significant correlation. Based on this finding, preoperative joint width rather than flexion contracture of knee seems to be more appropriate to represent the severity of arthritis. In the study conducted by Katie Rooks, they found out that the severity of preoperative radiographic arthritis (K-L grading scale) was associated with a higher degree of satisfaction [20]. This conclusion was in accordance with our result as well. For patients with pre-mJSW < 2 mm, the proportion in satisfied group was much more than that in dissatisfied group (64.6% vs 43.3%, p < 0.05). The improvement in measurement index of pain, which was the NRS in our study, was positively correlated with satisfaction prominently. However, the NRS-Rest change showed no significant difference between the two groups. In a multi-center prospective study conducted by F. Merle-Vincent et al., age older than 70 years at surgery was associated with a higher satisfaction rate [3]. Nevertheless, the proportion of patients older than 65 years was statistically higher in dissatisfied group in our study, compared with satisfied group (83.9% vs 57.7%, p < 0.05). In many previous studies, greater functional impairments or less function relief had been shown in older patients [21–23]. Patients with older age are more apt to be affected by comorbidities that are likely to limit their function rehabilitation after surgery in the long term. What is more, a recent study found that patients ≥ 68 years were more likely to experience MCID deterioration ≥ 7 points in functional outcomes and quality of life from 2 to 10 years after TKA [24].

Among the factors whose changes might correlate with patients’ satisfaction in our study, the varus angle of knee (radiological parameter) and preoperative limb deformity were rarely studied before. The varus angle change revealed statistical significance according to the level of satisfaction, with a better varus deformity correction accompanied by a higher degree of satisfaction. The same results can be supported by the study conducted by Shuichi Matsuda, and they pointed out that an important way to increase satisfaction was to achieve proper postoperative knee alignment [23]. Another interesting conclusion from our study was an inverse correlation between satisfaction and education level. Few studies explored this connection before. However, combined with the results from the study by Kristie B., one possible explanation for the inverse correlation was that patients with inadequate health literacy had “lower” overall expectations before surgery compared with adequate health literacy group [25]. Thus, their “lower” expectations were more likely to be achieved.

Our study demonstrated the MCIDs for the HSS and the NRS-Walking score to be 5.41 and 1.24 by simple linear regression model. The MCID was calculated between patients with just “satisfied” and those with just “acceptable” with the anchor-based method. Anchor-based method determines the MCID by associating the changes in the numerical scale for an outcome to some other subjective and independent assessments of improvement [14]. Plenty of previous studies demonstrated the MCIDs in OKS, KSS, VAS, WOMAC, and etc. In this study, we focused on the traditional but more conveniently used clinical parameters: HSS score and NRS score. Similar as the “anchor-based” method adopted by N.D. Clement [13], we address that MCID is a patient-centered concept, capturing both the improvements of the parameters and the feelings patients place on the change. Our results only indicate that patients achieving the MCID in objective parameter are associated with greater likelihood of getting satisfied after the procedure.

Additionally, according to the ROC curve analysis, we located the MIC as the threshold (cut-off point) for HSS score and NRS-Walking score, indicating the smallest change from baseline clinically perceived by patient in cohort. The MIC for HSS score was 25.5 and for NRS-Walking score was 6.5. The AUC (area under curve) of both parameters could be considered as prominent discrimination [26]. According to the cut-off point of ROC curve, we desired to find a threshold of improvement that enabled the statistical significance equating to the clinical satisfaction for patients. We also need to be aware of the fact that a patient-reported outcome like the NRS may not have a single MCID or MIC, and values for the MCIDs vary depending on the intervention and method we apply. The MICs and MCIDs in this study can help clinicians and surgeons with the interpretations of within-group changes (MIC) and between-group changes (MCID) 3 years after TKA [27].

We had several limitations in this study: first of all, the number of patients we enrolled is relatively small. Actually, there is no consensus on the minimum sample size required to determine a MCID or MIC up to now [28, 29]. Nevertheless, the calculation of MCID is performed according to patients with just “satisfied” and those with just “acceptable.” When correctly calculated by this specific method of MCID, the number of samples to be compared is 42 (satisfied) and 25 (acceptable), respectively. And under this circumstance, our relatively small sample size is no longer fatal for this kind of study.

Secondly, we applied the anchor-based method to calculate MCID in our study. However, patient satisfaction is difficult to evaluate precisely, and there is no gold standard for measuring it. Naturally, limited by the choice of the anchor (patient’s satisfaction) which is a subjective assessment, the results may be susceptible to bias. Besides, the MIC causing the differences in the scoring and satisfaction rate may be somewhat affected by subjective factors. As we mentioned above, satisfaction could be a complicated index that might be largely influenced by the quality of medical service or the delivery of humanistic care. To sum up, patient’s satisfaction is not solely a reflection of surgical outcome and should be interpreted with caution.

Thirdly, limited by the characteristics of retrospective pilot study, the demographics of our cohort may not represent the overall Chinese population undergoing primary TKA. Nevertheless, this pilot study is just an exploration for the next-step cohort study which is going to enroll patients all over the country with more representative features.

Finally, we did not record the comorbidity of patients at baseline. Some comorbidities can cause negative effects on the postoperative function improvements, especially for the elders. Therefore, these confounding factors may lead to the bias when evaluating clinical parameters, such as postoperative HSS score.

## Conclusion

We identified several factors that correlated with patients’ satisfaction after TKA in a long-term. In addition, we revealed the minimal clinically important difference (MCID) and minimum important change (MIC) for HSS and NRS score. The value of our study is to identify the minimal improvement in clinical parameter that is capable of predicting patient’s satisfaction after the procedure.

## Supplementary Information

**Additional file 1.** Supplementary Material 1 Preoperative minimal joint space width (pre-mJSW) illustrated in X-rays. Supplementary Material 2 Preoperative alignment measurement (varus angle of knee) illustrated in X-rays.

## Data Availability

The datasets used and/or analyzed during the current study are available from the corresponding author on reasonable request.

## Abbreviations

TKA: Total knee arthroplasty
MCID: Minimal clinically important difference
MIC: Minimum important change
OA: Osteoarthritis
HSS: Hospital special surgery
NRS: Numerical rating scales
VAK: Varus angle of knee
Pre-mJSW: Preoperative minimal joint space width
BMI: Body mass index
CI: Confidence interval
