# Supplementary material for: How much improvement can satisfy patients? Exploring patients’ satisfaction 3 years after total knee arthroplasty
Source: J Orthop Surg Res. 2021 Jun 17;16:389. doi: 10.1186/s13018-021-02514-2 (PMC8212506; doi:10.1186/s13018-021-02514-2)
Supplement: Supplementary file 1 — Additional file 1. Supplementary Material 1 Preoperative minimal joint space width (pre-mJSW) illustrated in X-rays. Supplementary Material 2 Preoperative alignment measurement (varus angle of knee) illustrated in X-rays. [file 13018_2021_2514_MOESM1_ESM.docx]

**Supplementary Materials**


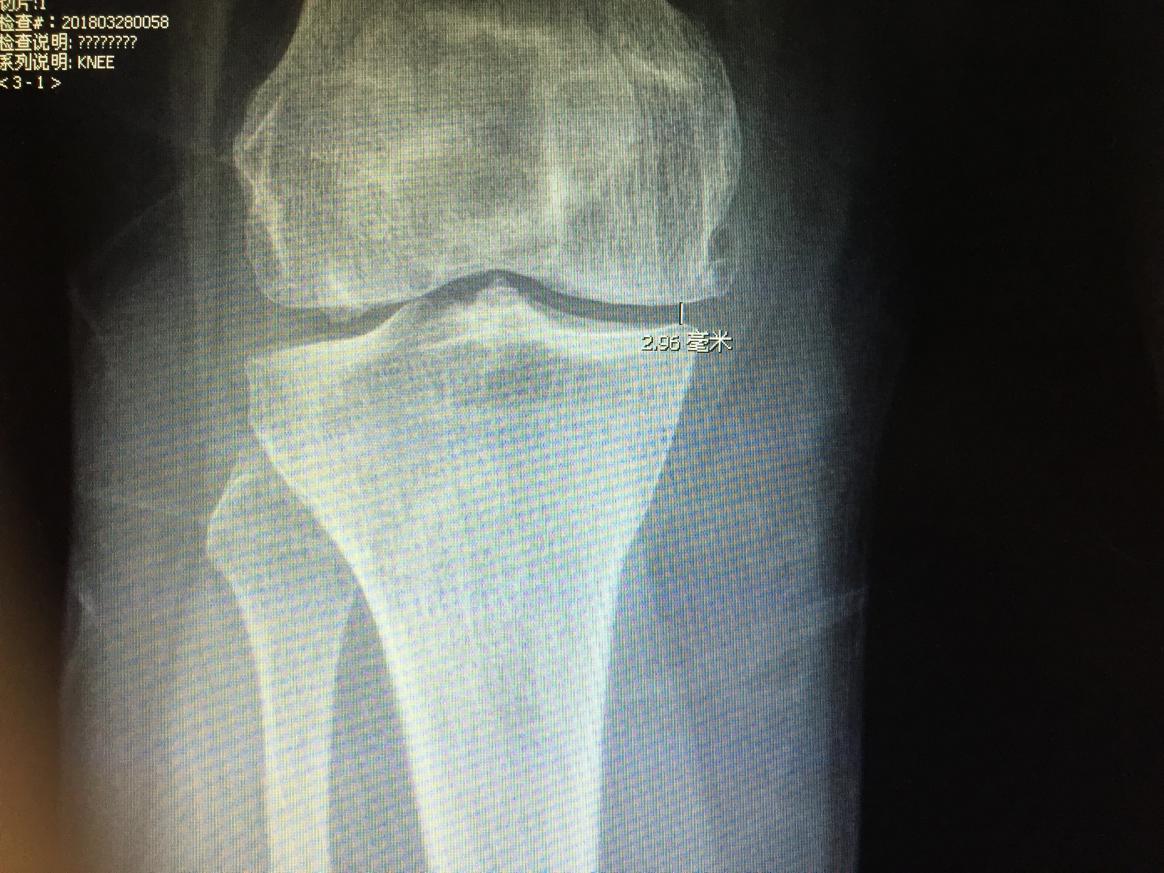


Material 1 Preoperative minimal joint space width(pre-mJSW)illustrated in X-rays


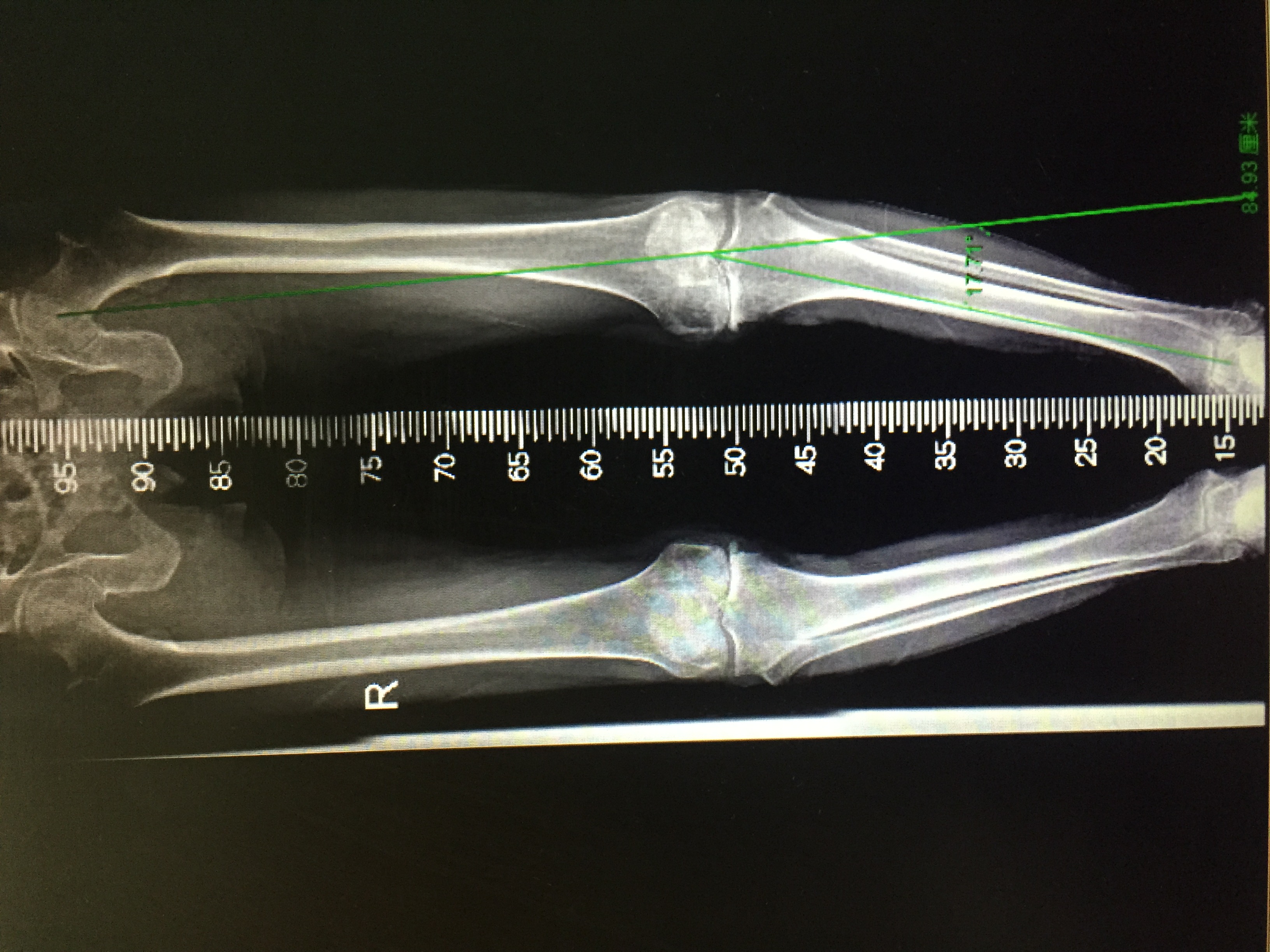


Material 2 Preoperative alignment measurement(varus angle of knee)illustrated in X-rays
